# Brønsted Acidic Ionic Liquid Accelerated Halogenation of Organic Compounds with *N*-Halosuccinimides (NXS)

**DOI:** 10.3390/molecules18010074

**Published:** 2012-12-21

**Authors:** Dejan Vražič, Marjan Jereb, Kenneth K. Laali, Stojan Stavber

**Affiliations:** 1 Laboratory for Organic and Bioorganic Chemistry, Jožef Stefan Institute, Jamova 39, 1000 Ljubljana, Slovenia; 2 Faculty for Chemistry and Chemical Technology, University of Ljubljana, Aškerčeva 5, 1000 Ljubljana, Slovenia; 3 Department of Chemistry, University North Florida, Science and Engineering Building, 1 UNF Drive, Jacksonville, FL 32224, USA; 4 Centre of Excellence for Integrated Approaches in Chemistry and Biology of Proteins CIPKeBiP, Jamova 39, 1000 Ljubljana, Slovenia

**Keywords:** Brønsted-acidic ionic liquid, halogenation, aromatic ketones, NXS reagents, green chemistry

## Abstract

The Brønsted-acidic ionic liquid 1-methyl-3-(4-sulfobutyl)imidazolium triflate [BMIM(SO_3_H)][OTf] was demonstrated to act efficiently as solvent and catalyst for the halogenation of activated organic compounds with *N*-halosuccinimides (NXS) under mild conditions with short reaction times. Methyl aryl ketones were converted into α-halo and α,α-dihaloketones, depending on the quantity of NXS used. Ketones with activated aromatic rings were selectively halogenated, however in some cases mixtures of α-halogenated ketone and ring-halogenated ketones were obtained. Activated aromatics were regioselectively ring halogenated to give mono- and dihalo-substituted products. The [BMIM(SO_3_H)][OTf] ionic liquid (IL-A) was successfully reused eight times in a representative monohalogenation reaction with no noticeable decrease in efficiency. An effective halogenation scale-up in this IL is also presented. The reactivity trend and the observed chemo- and regioselectiivities point to an ET process in these IL-promoted halofunctionalization reactions.

## 1. Introduction

Halogenated organic compounds are highly versatile starting materials and intermediates in organic and organometallic chemistry and their production has been under constant investigation. The topic has been extensively reviewed [1,2,3,4]. Halogenation reactions are often associated with extensive waste production and relatively high process costs. Increasing environmental and climate changes urge the development of safer and “greener” synthetic pathways at reduced costs. The main reaction waste is usually the organic solvent used as reaction medium; moreover volatile organics have severe environmental and health issues. 

*N*-halosuccinimides are very popular as halogen-transfer reagents [5,6,7,8,9]; however they are usually not reactive enough for direct halogenation and require a Lewis acid or a Brønsted acid catalyst. The catalysts employed are usually moisture sensitive, they are often metallic or strongly acidic, produce toxic waste, and are costly. 

**Figure 1:**
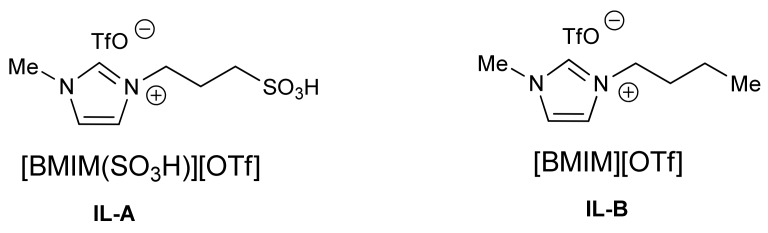
Structure of investigated imidazolium-based ionic liquids IL-A and IL-B.

Halogenation of aromatic compounds in different ILs has been recently reviewed [10,11] and further studied [12]. Fluorination of arenes with F-TEDA-BF_4_ was examined in imidazolium-ILs [13]. Other earlier studies include chlorination of aromatics with trichloroisocyanuric acid in IL-A (Figure 1) [14], oxidative chlorination with HCl in [Hmim][NO_3_] [15], bromination of aromatics in tribromide-based ILs [16,17,18,19], iodination with I_2_/H_2_O_2_ in BMIM-IL [20], I_2_/F-TEDA-BF_4_ in imidazolium- and pyridinium-IL, also with I_2_ or MeI/Koser’s reagent in BMIM-IL [21], iodination of alcohols in [Hmim][HSO_4_] [22], and with *N*-iodosaccharin [23].

Brønsted-acidic ionic liquids such as [BMIM(SO_3_H)][OTf] [24] (IL-A) have attracted considerable attention as dual solvent/catalysts and were employed in diverse acid-catalyzed transformations [25,26,27,28,29,30].

In continuation of our previous studies on application of ionic liquids as solvents and catalysts in synthesis [31,32,33,34,35,36,37,38,39,40,41,42,43], we report on the utility of IL-A for halofunctionalization of organic molecules with NXS reagents.

## 2. Results and Discussion

Halogenation of acetophenone (**1**) with NIS, NBS and NCS was studied in IL-A and IL-B (Figure 1), and α-halogenation took place with all three reagents (Table 1). Iodination of **1** with 1.1 equiv. of NIS at 55 °C gave **2a** with significantly higher conversion in IL-A than in IL-B; albeit with slightly lower selectivity (Table 1, entries 1 and 2). Similar results were obtained in bromination with NBS (entries 3 and 4). Bromination with 2.2 equiv. of NBS in IL-A resulted in quantitative dibromination, whereas in IL-B only monobromination took place (entries 5 and 6). Chlorofunctionalization with 1.1 equivalents of NCS in the both IL-A and IL-B gave **2c** as the main product; whereas regioselectivity in IL-A changed after prolonged heating and **3c** was obtained as the mayor product (entries 7–9). Reaction with 2.2 equiv. of NCS in IL-B gave **2c** as major and **3c** as minor products, whereas **3c** was formed exclusively in IL-A (entries 10 and 11). The results confirm the superior catalytic effect of the Brønsted acidic IL-A.

Focusing on the effect of the structure of aryl methyl ketones **4** on halogenation with *N*-halosuccinimides in IL-A (Table 2), compound **4a** was selectively converted into its α-iodo derivative **5a** (entry 1). Functionalization of **4a** with 1.1 equiv. of NBS was less selective, furnishing α-bromo- and α,α-dibromo- substituted ketones **6a** and **7a** (entry 2), while reaction with 2.2 equiv of NBS yielded **7a** in excellent yield (entry 3). Chlorination with 2.2 equiv. of NCS selectively produced the α,α-dichloroketone **8a** (entry 4). 

Halogenation of 2-acetylthiophene (**4b**) yielded the α-iodoketone **5b** regioselectively (entry 5). Reaction of **4b** with NBS (1.1 equiv.) exhibited a similar level of selectivity as with **4a**, forming the mono- and dibrominated products **6b** and **7b** (entry 6), and with 2.2 equiv. of NBS **7b** in high yield. Functionalization with 2.2 equiv. of NCS selectively furnished the α,α-dichloroketone **8b** (entry 8). Compound **4c** was regioselectively converted to its α-iodoketone **5c** with NIS in high yield (entry 9). Bromination with NBS was somewhat less selective than in the previous cases giving a mixture of α-bromo and α,α-dibromoketones **6c** and **7c** (entry 10), while with 2.2 equiv. of NBS **7c** was selectively obtained in high yield (entry 11). The reaction temperatures were in 55–70 °C range, depending on the reactivity of the NXS reagent, and the reaction times varied from 10 to 150 minutes. It is noteworthy that no ring functionalization products were formed with ketones **4a**, **4b** and **4c**, despite the additional activation of the aromatic ring with an EDG functionalization, which was obviously not enough for acetyl group deactivation of the aromatic part of the target. 

**Scheme 1:**
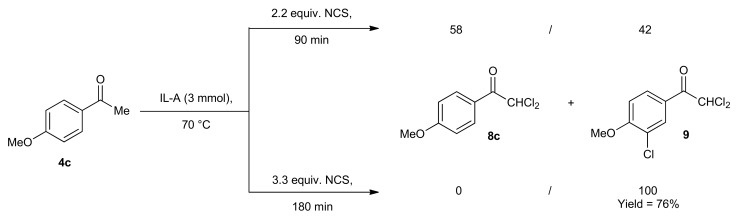
Chlorination of 4′-methoxyacetophenone (**4c**) with NCS.

Halofunctionalization of **4c** with excess of NCS was studied in IL-A at 70 °C (Scheme 1). With 2.2 equiv. of NCS a mixture of 2,2-dichloro-1-(*p*-anisyl)ethanone (**8c**) and 2,2-dichloro-1-(3-chloro-4-methoxyphenyl)ethanone (**9**) was obtained in 58:42 ratio, respectively, and with 3.3 equiv. of NCS selectively compound **9** was formed in high yield as the sole product. 

**Scheme 2:**
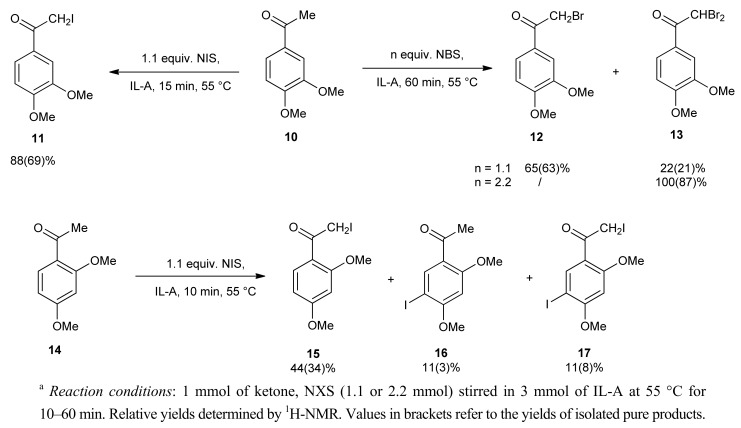
Halofunctionalization of 3′,4′-dimethoxyacetophenone (**10**) and 2′,4′-dimethoxyacetophenone (**14**) with NXS reagents in [BMIM(SO_3_H)][OTf] (IL-A) medium ^a^.

**Scheme 3:**
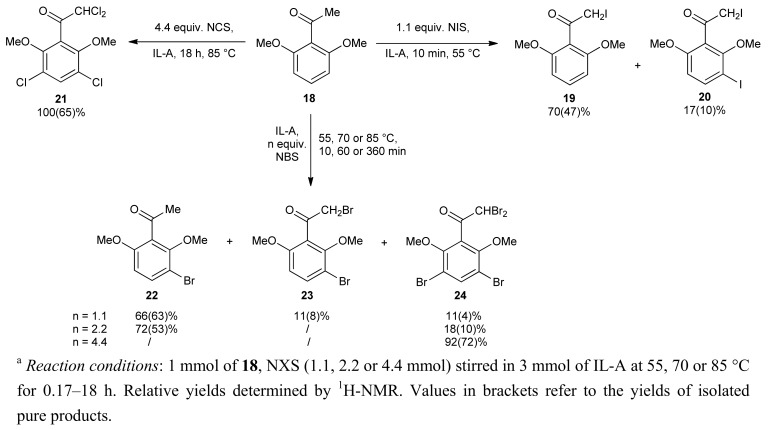
Halofunctionalization of 2′,6′-dimethoxyacetophenone (**18**) with NXS reagents in [BMIM(SO_3_H)][OTf] (IL-A) medium ^a^.

**Scheme 4:**
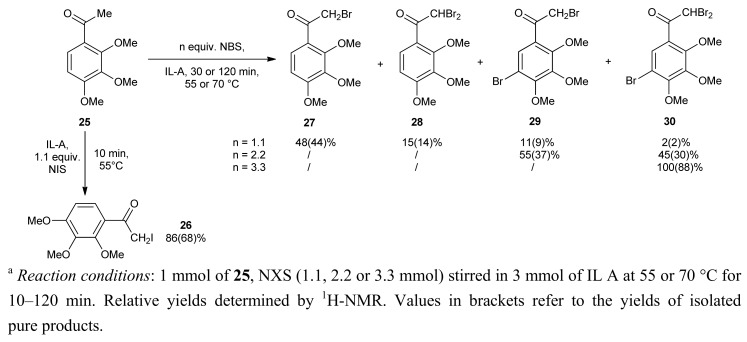
Halofunctionalization of 2′,3′,4′-trimethoxyacetophenone (**25**) with NXS reagents in [BMIM(SO_3_H)][OTf] (IL-A) medium ^a^.

In the next phase of this study halofunctionalization of isomeric dimethoxyacetophenones **10**, **14**, **18** and trimethoxyacetophenone **25** were examined (Scheme 2, Scheme 3 and Scheme 4). Transformation of **10** with 1.1 equiv. of NBS furnished a mixture of α-bromoketone **12** and α,α-dibromo derivative **13**, whereas with 2.2 equivalents of NBS, **13** was obtained as a sole product in high yield. Compound **10** was regioselectively converted into the α-iodoketone **11** with 1.1 equiv. NIS in good yield (Scheme 2).

Isomeric 2′,4′-dimethoxyacetophenone (**14**) exhibited different selectivity in reaction with 1.1 equiv. of NIS, forming a mixture of the α-iodoketone **15** as the major product along with 1-acetyl-5-iodo-2,4-dimethoxybenzene (**16**) and 2-iodo-1-(5-iodo-2,4-dimethoxyphenyl)ethanone (**17**) as minor products (Scheme 2). 2′,6′-Dimethoxyacetophenone (**18**) exhibited a similar reactivity trend as **14**. Functionalization of **18** with 4.4 equiv. of NCS in IL-A selectively furnished 2,2-dichloro-1-(3,5-dichloro-2,6-dimethoxyphenyl)ethanone (**21**) in a good yield (Scheme 3). Reaction of **18** with 1.1 equiv. of NIS furnished a mixture of α-iodoketone **19** as major, and 2-iodo-1-(3-iodo-2,6-dimethoxyphenyl)ethanone (**20**) as minor product. Functionalization of **18** with 1.1 equiv. of NBS yielded the 3-bromo derivative **22** (as the main product), along with 2-bromo-1-(3-bromo-2,6-dimethoxyphenyl)ethanone (**23**) and 2,2-dibromo-1-(3,5-dibromo-2,6-dimethoxyphenyl)ethanone (**24**) as minor products. A similar reactivity trend was observed with 2.2 equiv. of NBS, forming **22** and **24** (but **23** was not formed). When 4.4 equiv. of NBS was employed, the tetrabromo-derivative **24** was isolated as the sole product in good yield. 

2′,3′,4′-Trimethoxyacetophenone (**25**) was rapidly (within minutes) converted to the α-iodo derivative **26** with 1.1 equiv. of NIS (Scheme 4). Reaction of **25** with NBS gave a mixture of up to four compounds, depending on the quantity of the NBS. With 1.1 equiv. of NBS the α-bromoketone **27** was the main product, and α,α-dibromoketone **28**, 2-bromo-1-(5-bromo-2,3,4-trimethoxyphenyl)ethanone (**29)** and tribromo-substituted ketone **30** were the minor products. With 2.2 equiv. of NBS **29** and **30** were the only isolated products (**27** and **28** were not observed). Reaction of **25** with 3.3 equiv. of NBS exclusively yielded **30** in excellent yield (see Scheme 4).

**Scheme 5:**
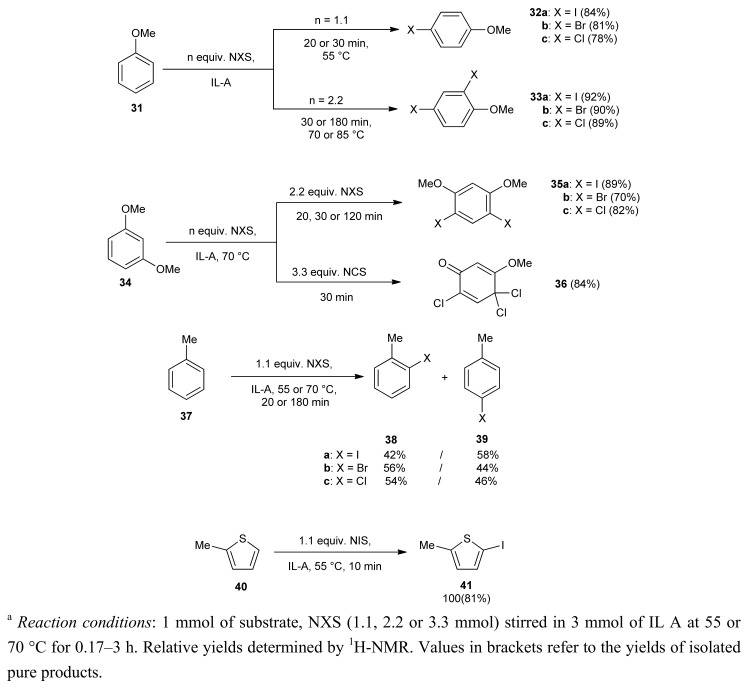
Transformation of activated aromatic molecules with *N*-halosuccinimides (NXS) in [BMIM(SO_3_H)][OTf] (IL-A) ^a^.

To explore the potential utility of NXS/IL-A system in ring halogenation of activated arenes, halofunctionalization of anisole (**31**), 1,3-dimethoxybenzene (**34**), toluene (**37**), and 2-methylthiophene (**40**) were examined (Scheme 5).

Reaction of anisole (**31**) with 1.1 equiv. of NXS was highly *para*-selective, forming the corresponding iodo- **32a**, bromo- **32b** and chloro-derivative **32c** (in 84-78% isolated yields) with no *ortho*-substitution being observed. Reaction of **31** with 2.2 equiv. of NXS selectively produced 2,4-dihaloanisoles **33a**–**c** in high yields. 

**Figure 2:**
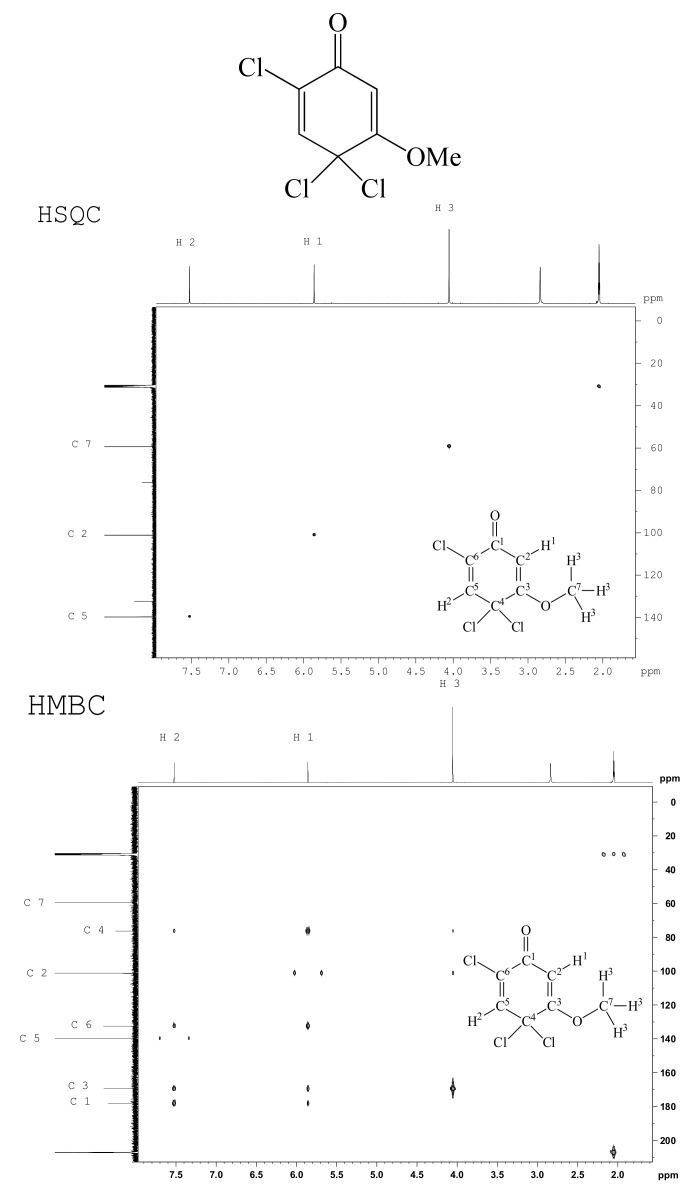
Copies of 2D-NMR spectra for 2,4,4-trichloro-5-methoxycyclohexa-2,5-dienone (**36**).

1,3-Dimethoxybenzene (**34**) was transformed with 2.2 equiv. of NIS, NBS and NCS into 2,4-dihalo-1,5-dimethoxybenzenes **35a**–**c** in high yields. Transformation of **34** with 3.3 equiv. of NCS led to a dearomatization of the target molecule and the formation of 2,4,4-trichloro-5-methoxycyclohexa-2,5-dienone (**36**) in 84% yield. This compound is the result of chlorination of aromatic ring resulting primarily in **35c** and further regioselective 1,4-addition-elimination process following *ipso-*chlorination and demethylation of the *para-*methoxy group, what was confirmed by an independent experiment and the structure of **36** unequivocally distinguished from the possibly formed isomeric 4,6,6-trichloro-3-methoxycyclohexa-2,4-dienone using 2D-NMR measurement techniques HSQC and HMBC (Figure 2). The also expected 1,3,5-trichloro-2,4-dimethoxybenzene was not formed probably because of substantial sterical hindrance between the two methoxy substituents on the aromatic ring. 

Halogenation of toluene (**37**) with 1.1 equiv. of NXS gave 4- and 2-halotoluene. The *ortho*/*para* ratio with NIS was 42:58, whereas with NBS and NCS this ratio changed in favor of the *ortho-*isomer. A similar regioselectivity was reported in halogenation of toluene with NBS and NCS in the presence of FeCl_3_ [5].

2-Methylthiophene (**40**) was selectively converted into its 5-iodo derivative **41** in high yield with 1.1 equiv. of NIS. Reaction of **40** with NCS and NBS were also carried out successfully, however the isolated yields were low due to the volatility of the products.

Having discovered these highly efficient halofunctionalization reactions we then explored the possibility to make the process more economical through recycling and reuse of the IL. To explore this possibility, halogenation of acetophenone (**1**) with 1.1 equiv. of NIS was selected as a prototype reaction and the reaction was successfully repeated in reused/recycled IL-A for 8 cycles (Table 3) with no notable decrease in the conversions. To address the question of relative reactivity of the NXS reagent versus recycling and reuse, bromofunctionalization of **1** with 2.2 equivalents of NBS was examined (Table 3) over 5 repeated cycles, showing a gradual decrease in the dibromo derivative **3b** with concomitant increase in monobromo product **2b**. The data underscores the importance of IL-A as a promoter in these halogen-transfer reactions. 

Finally to explore the feasibility to perform these reactions on larger scales than reported thus far, bromination of **1** was carried out on a 9 mmol scale (a 9 fold increase), by using 19.8 mmols of NBS, and 27 mmol of IL-A. The 2,2-dibromo-1-phenylethanone **3b** was obtained in 90% isolated yield after 40 minutes at 70 °C. 

Concerning a plausible mechanism for halofunctionalization with NXS/[BMIM(SO_3_H)][OTf] systems, previous studies [44,45,46,47] have shown that ring halogenations of activated aromatics with NIS, NBS and NCS proceed through an electron transfer (ET) pathway as the main reaction channel. Based on the fact that in the present study under the reaction conditions employed only activated aromatics could be halogenated, and considering the observed chemo- and regioselectivity patterns, in all probability the ET reaction pathway is also most likely operating in these reactions. The role of acidic IL as a catalyst is to enhance the electrophilic character of NXS reagents by additional polarization of N-X bond. In halogenation of phenyl methyl ketones the acidic IL could also act as enolization catalyst, accelerating electrophilic addition of halogen. With the studied ketones, side chain halogenation is the primary event with ring halogenation manifesting only when the ring is highly activated (two methoxy groups). 

## 3. Experimental

### 3.1. General

All chemicals were purchased from commercial sources and used without further purification. Column chromatography was carried out using silica gel 60 (particle size: 0.063–0.2 mm) and preparative thin layer chromatography using PLC Silica gel 60 F_254_, 2 mm plates. Melting points were obtained with a Büchi 535 apparatus. For obtaining IR spectra on FTS3000-MX spectrometer, either a KBr pellet of the product was made or NaCl plates were used based on the physical state of product. NMR spectra were recorded on a Bruker Avance 300 DPX instrument (^1^H: 300 MHz, ^13^C: 75.5 MHz). The ^1^H spectra were referred to an internal standard (0 ppm for TMS) or to the residual ^1^H signal of CHCl_3_ at 7.26 ppm and for CD_3_COCD_3_ at 2.05 ppm (central line). The ^13^C spectra were referred to the residual signal of CDCl_3_ (77.0 ppm, central line) or CD_3_COCD_3_ (30.8 ppm, central line). Elemental analysis was performed on a Perkin-Elmer 2400-Series 2 apparatus.

### 3.2. Representative Procedure for Halogenation of Aromatic Compounds

To a stirred mixture of the aromatic compound (1 mmol) in ionic liquid (3 mmol), *N*-halo-succinimide (1.1–4.4 mmol) was added. The reaction mixture was stirred at 55, 70 or 85 °C until completion (monitored by TLC). The mixture was cooled to room temperature and washed three times with dichloromethane. The combined organic fractions were washed with aqueous Na_2_S_2_O_3_ and NaHCO_3_, dried over anhydrous Na_2_SO_4_ and filtered. Solvent was removed under reduced pressure and the crude reaction mixture was analyzed by ^1^H-NMR. Isolation of products from reaction mixtures was accomplished by preparative TLC or column chromatography and pure compounds were analyzed by NMR, MS and IR spectroscopy. Data for known compounds were verified from the literature while the new compounds were completely verified by spectroscopic data and C, H, N elemental analysis. 

*2-Iodo-1-phenylethanone* (**2a**). One mmol of substrate, 3 mmol IL-A, 1.1 mmol NIS, r.t. = 10 min, 55 °C, preparative chromatography SiO_2_, hexane/CH_2_Cl_2_ = 2.5/7.5, orange oil (86%), mp: lit [48] 33–35 °C; ^1^H-NMR (CDCl_3_): δ 8.03–7.97 (m, 2H), 7.64–7.57 (m, 1H), 7.54–7.45 (m, 2H), 4.37 (s, 2H); IR(NaCl): 3059, 2976, 2938, 1674, 1595, 1449, 1418, 1271, 1173, 1105, 1001, 791, 747, 703 cm^−1^; MS (ESI): 247.0 (M+H)^+^.

*2-Bromo-1-phenylethanone* (**2b**). One mmol of substrate, 3 mmol IL-A, 1.1 mmol NBS, r.t. = 20 min, 55 °C), preparative chromatography, SiO_2_, hexane/CH_2_Cl_2_ = 2.5/7.5, white solid (73%), mp: 46.8–48.2 °C (lit [49] 48.8–49.3 °C); ^1^H-NMR (CDCl_3_): δ 8.02–7.97 (m, 2H), 7.66–7.57 (m, 1H), 7.55–7.45 (m, 2H), 4.46 (s, 2H); IR (KBr): 3058, 3002, 2951, 1694, 1593, 1447, 1390, 1281, 1198, 991, 881, 746, 685, 623 cm^−1^; MS (ESI): 199.0 (M+H)^+^, 201.0 (M+2+H)^+^.

*2-Chloro-1-phenylethanone* (**2c**). One mmol of substrate, 3 mmol IL-B, 1.1 mmol NCS, r.t. = 30 min, 70 °C, preparative chromatography SiO_2_, hexane/CH_2_Cl_2_ = 2.5/7.5, white solid (74%), mp: 53.2–54.2 °C (lit [50] 55 °C); ^1^H-NMR (CDCl_3_): δ 8.00–7.93 (m, 2H), 7.66–7.58 (m, 1H), 7.55–7.46 (m, 2H), 4.71 (s, 2H); IR (neat): 2951, 1692, 1595, 1580, 1448, 1397, 1209, 999, 769, 746, 685, 640 cm^−1^; MS (ESI): 155.0 (M+H)^+^, 157.0 (M+2+H)^+^.

*2,2-Dibromo-1-phenylethanone* (**3b**). One mmol of substrate, 3 mmol IL-A, 2.2 mmol NBS, r.t. = 30 min, 70 °C, CC SiO_2_, CH_2_Cl_2_, white solid (82%), mp: 31.5–32.9 °C (lit [51] 33.5–34.5 °C); ^1^H-NMR (CDCl_3_): δ 8.13–8.04 (m, 2H), 7.69–7.60 (m, 1H), 7.57–7.46 (m, 2H), 6.70 (s, 1H); IR (KBr): 1693, 1587, 1445, 1267, 1198, 983, 797, 705, 678, 625 cm^−1^; MS (ESI): 276.9 (M+H)^+^, 278.9 (M+2+H)^+^, 280.9 (M+4+H)^+^.

*2,2-Dichloro-1-phenylethanone* (**3c**). One mmol of substrate, 3 mmol IL-A, 2.2 mmol NCS, r.t. = 120 min, 70 °C, CC SiO_2_, CH_2_Cl_2_, yellow oil [52] (96%); ^1^H-NMR (CDCl_3_): δ 8.13–8.06 (m, 2H), 7.69–7.62 (m, 1H), 7.57–7.49 (m, 2H), 6.68 (s, 1H); IR (NaCl): 3065, 3008, 1705, 1595, 1449, 1278, 1222, 1182, 990, 932, 810, 774, 685, 654 cm^−1^; MS (ESI): 154.0 (M−Cl+H)^+^, 156.0 (M−Cl+2+H)^+^.

*2-Iodo-1-(2-naphthyl)ethanone* (**5a**). One mmol of substrate, 3 mmol IL-A, 1.1 mmol NIS, r.t. = 10 min, 55 °C, preparative chromatography SiO_2_, hexane/CH_2_Cl_2_ = 2.5/7.5, yellow solid (84%), mp: 87.5–90.2 °C (lit [53] 87–87.5 °C); ^1^H-NMR (CDCl_3_): δ 8.54–8.51 (m, 1H), 8.06–7.96 (m, 2H), 7.94–7.87 (m, 2H), 7.67–7.54 (m, 2H), 4.49 (s, 2H); IR(KBr): 1661, 1622, 1464, 1368, 1285, 1231, 1144, 1123, 1094, 1020, 991, 866, 804, 750 cm^−1^; MS (ESI): 297.0 (M+H)^+^.

*2-Iodo-1-(2-thienyl)ethanone* (**5b**). One mmol of substrate, 3 mmol IL-A, 1.1 mmol NIS, r.t. = 10 min, 55 °C, preparative chromatography SiO_2_, hexane/CH_2_Cl_2_ = 2.5/7.5, yellow oil [54] (80%); ^1^H-NMR (CDCl_3_): δ 7.80 (dd, *J* = 3.9 Hz, *J* = 1.0 Hz, 1H), 7.69 (dd, *J* = 4.9 Hz, *J* = 1.0 Hz, 1H), 7.16 (dd, *J* = 4.9 Hz, *J* = 3.9 Hz, 1H), 4.30 (s, 2H); IR (NaCl): 3089, 3036, 2972, 2936, 1650, 1514, 1412, 1355, 1280, 1164, 1099, 1061, 963, 858, 727 cm^−1^; MS (ESI): 252.9 (M+H)^+^.

*1-(p-Anisyl)-2-iodoethanone* (**5c**). One mmol of substrate, 3 mmol IL-A, 1.1 mmol NIS, r.t. = 10 min, 55 °C, preparative chromatography SiO_2_, hexane/CH_2_Cl_2_ = 2.5/7.5, grey solid (80%), mp: 56.3–59.4 °C (lit [48] 57–59.5 °C); ^1^H-NMR (CDCl_3_): δ 7.97 (d, *J* = 8.9 Hz, 2H), 6.95 (d, *J* = 8.9 Hz, 2H), 4.31 (s, 2H), 3.89 (s, 3H); IR (KBr): 3032, 2972, 1656, 1601, 1568, 1508, 1454, 1422, 1288, 1257, 1173, 1099, 1016, 1001, 845, 754 cm^−1^; MS (ESI): 277.0 (M+H)^+^.

*2-Bromo-1-(2-naphthyl)ethanone* (**6a**). One mmol of substrate, 3 mmol IL-A, 1.1 mmol NBS, r.t. = 20 min, 55 °C, preparative chromatography SiO_2_, hexane/CH_2_Cl_2_ = 2.5/7.5, grey solid (66%), mp: 80.3–82.2 °C (lit [55] 78–79.5 °C); ^1^H-NMR (CDCl_3_): δ 8.54–8.50 (m, 1H), 8.07–7.96 (m, 2H), 7.96–7.87 (m, 2H), 7.68–7.54 (m, 2H), 4.58 (s, 2H); IR (KBr): 2999, 2948, 1690, 1624, 1591, 1468, 1385, 1175, 1159, 1127, 1028, 853, 812, 740, 680 cm^−1^; MS (ESI): 249.0 (M+H)^+^, 251.0 (M+2+H)^+^.

*2-Bromo-1-(2-thienyl)ethanone* (**6b**). One mmol of substrate, 3 mmol IL-A, 1.1 mmol NBS, r.t. = 10 min, 55 °C, preparative chromatography SiO_2_, hexane/CH_2_Cl_2_ = 2.5/7.5, yellow oil (72%), mp: lit [56] 31–33 °C; ^1^H-NMR (CDCl_3_): δ 7.81 (dd, *J* = 3.9 Hz, *J* = 1.0 Hz, 1H), 7.72 (dd, *J* = 4.9 Hz, *J* = 1.0 Hz, 1H), 7.17 (dd, *J* = 4.9 Hz, *J* = 3.9 Hz, 1H), 4.36 (s, 2H); IR (NaCl): 3098, 2942, 1652, 1514, 1412, 1355, 1287, 1194, 1110, 1061, 973, 939, 854, 727, 667 cm^−1^; MS (ESI): 204.9 (M+H)^+^, 206.9 (M+2+H)^+^.

*2-Bromo-1-(p-anisyl)ethanone* (**6c**). One mmol of substrate, 3 mmol IL-A, 1.1 mmol NBS, r.t. = 20 min, 55 °C, preparative chromatography SiO_2_, hexane/CH_2_Cl_2_ = 2.5/7.5, pale red (62%), mp: 68.4–69.7 °C (lit [56] 69–71 °C); ^1^H-NMR (CDCl_3_): δ 7.97 (d, *J* = 8.9 Hz, 2H), 6.96 (d, *J* = 8.9 Hz, 2H), 4.40 (s, 2H), 3.89 (s, 3H); IR (KBr): 2938, 1688, 1597, 1510, 1326, 1263, 1208, 1167, 1115, 1016, 986, 745, 685 cm^−1^; MS (ESI): 229.0 (M+H)^+^, 231.0 (M+2+H)^+^.

*2,2-Dibromo-1-(2-naphthyl)ethanone* (**7a**). One mmol of substrate, 3 mmol IL-A, 2.2 mmol NBS, r.t. = 60 min, 70 °C, CC SiO_2_, CH_2_Cl_2_, white solid (94%), mp: 78.1–80.7 °C (lit [57] 100–101.5 °C); ^1^H-NMR (CDCl_3_): δ 8.67–8.63 (m, 1H), 8.13–8.07 (m, 1H), 8.04–7.87 (m, 3H), 7.70–7.56 (m, 2H), 6.86 (s, 1H); IR (KBr): 1682, 1620, 1587, 1358, 1273, 1182, 1153, 1119, 820, 733, 683, 623 cm^−1^; MS (ESI): 326.9 (M+H)^+^, 328.9 (M+2+H)^+^, 330.9 (M+4+H)^+^.

*2,2-Dibromo-1-(2-thienyl)ethanone* (**7b**). One mmol of substrate, 3 mmol IL-A, 2.2 mmol NBS, r.t. = 30 min, 70 °C, preparative chromatography SiO_2_, hexane/CH_2_Cl_2_ = 2.5/7.5, orange oil [58] (85%); ^1^H-NMR (CDCl_3_): δ 8.03–7.98 (m, 1H), 7.80–7.76 (m, 1H), 7.22–7.18 (m, 1H), 6.48 (s, 1H); IR (NaCl): 3098, 3013, 1669, 1512, 1411, 1354, 1279, 1179, 1065, 937, 858, 726, 665, 638 cm^−1^; MS (ESI): 282.9 (M+H)^+^, 284.9 (M+2+H)^+^, 286.8 (M+4+H)^+^.

*2,2-Dibromo-1-(p-anisyl)ethanone* (**7c**). One mmol of substrate, 3 mmol IL-A, 2.2 mmol NBS, r.t. = 30 min, 70 °C, CC SiO_2_, CH_2_Cl_2_, white solid (91%), mp: 86.7–88.9 °C (lit [51] 93–94 °C); ^1^H-NMR (CDCl_3_): δ 8.08 (d, *J* = 9.0 Hz, 2H), 6.98 (d, *J* = 9.0 Hz, 2H), 6.66 (s, 1H), 3.90 (s, 3H); IR(KBr): 3032, 1674, 1597, 1566, 1504, 1420, 1314, 1263, 1202, 1177, 1146, 1020, 982, 845, 810, 761, 706, 683 cm^−1^; MS (ESI): 306.9 (M+H)^+^, 308.9 (M+2+H)^+^, 310.9 (M+4+H)^+^.

*2,2-Dichloro-1-(2-naphthyl)ethanone* (**8a**). One mmol of substrate, 3 mmol IL-A, 2.2 mmol NCS, r.t. = 150 min, 70 °C, CC SiO_2_, CH_2_Cl_2_, yellow solid (86%), mp: 66.6–69.8 °C (lit [59] 80–80.5 °C); ^1^H-NMR (CDCl_3_): δ 8.68–8.62 (m, 1H), 8.13–8.06 (m, 1H), 8.04–7.87 (m, 3H), 7.71–7.56 (m, 2H), 6.83 (s, 1H); IR (KBr): 3059, 1699, 1626, 1596, 1466, 1357, 1282, 1219, 1175, 1125, 866, 824, 785, 742, 657 cm^−1^; MS (ESI): 239.0 (M+H)^+^, 241.0 (M+2+H)^+^.

*2,2-Dichloro-1-(2-thienyl)ethanone* (**8b**). One mmol of substrate, 3 mmol IL-A, 2.2 mmol NCS, r.t. = 90 min, 70 °C, preparative chromatography SiO_2_, hexane/CH_2_Cl_2_ = 3.5/6.5, orange oil [60] (67%); ^1^H-NMR (CDCl_3_): δ 8.02 (dd, *J* = 3.9 Hz, *J* = 1.0 Hz, 1H), 7.80 (dd, *J* = 4.9 Hz, *J* = 1.0 Hz, 1H), 7.21 (dd, *J* = 4.9 Hz, *J* = 3.9 Hz, 1H), 6.47 (s, 3H); IR (NaCl): 3100, 3007, 1682, 1512, 1409, 1356, 1277, 1240, 1217, 1188, 1067, 944, 858, 802, 720, 668 cm^−1^; MS (ESI): 194.9 (M+H)^+^.

*2,2-Dichloro-1-(p-anisyl)ethanone* (**8c**). One mmol of substrate, 3 mmol IL-A, 2.2 mmol NCS, r.t. = 90 min, 70 °C, preparative chromatography SiO_2_, hexane/CH_2_Cl_2_ = 3.5/6.5, white solid (36%), mp: 76.9–78.2 °C (lit [61] 78–79 °C); ^1^H-NMR (CDCl_3_): δ 8.08 (d, *J* = 8.9 Hz, 2H), 6.99 (d, *J* = 8.9 Hz, 2H), 6.63 (s, 1H), 3.90 (s, 3H); IR (KBr): 3028, 1683, 1600, 1568, 1506, 1422, 1318, 1298, 1265, 1236, 1175, 1024, 847, 795, 745, 646 cm^−1^; MS (ESI): 219.0 (M+H)^+^, 221 (M+2+H)^+^.

*2,2-Dichloro-1-(3-chloro-4-methoxyphenyl)ethanone* (**9**). One mmol of substrate, 3 mmol IL-A, 3.3 mmol NCS, r.t. = 180 min, 70 °C; preparative chromatography SiO_2_, hexane/CH_2_Cl_2_ = 2.5/7.5; white solid (76%); mp: 99.3–100.9 °C; ^1^H-NMR (CDCl_3_): δ 8.14 (d, *J* = 2.3 Hz, 1H), 8.05 (dd, *J* = 8.8 Hz, *J* = 2.3 Hz, 1H), 7.02 (d, *J* = 8.8 Hz, 1H), 6.57 (s, 1H), 4.00 (s, 3H); ^13^C-NMR (CD_3_COCD_3_): δ 185.7, 161.9, 133.1, 132.5, 127.0, 124.6, 114.3, 70.0, 58.2; IR (KBr): 2982, 1686, 1591, 1508, 1318, 1281, 1227, 1182, 1148, 1065, 1009, 824, 797, 750, 700, 631 cm^−1^; MS (ESI): 253.0 (M+H)^+^, 255.0 (M+2+H)^+^; HRMS: calcd for C_9_H_8_Cl_3_O_2_: 252.9590; found: 252.9601; Anal. Calcd for C_9_H_7_Cl_3_O_2_: C, 42.64; H, 2.78. Found: C, 42.92; H, 2.72.

*2-Iodo-1-(3,4-dimethoxyphenyl)ethanone* (**11**). One mmol of substrate, 3 mmol IL-A, 1.1 mmol NIS, r.t. = 15 min, 55 °C, preparative chromatography SiO_2_, CH_2_Cl_2_, yellow solid (69%), mp: 62.0–65.0 °C (lit [62] 65.2–66.2 °C); ^1^H-NMR (CDCl_3_): δ 7.62 (dd, *J* = 8.4 Hz, *J* = 2.0 Hz, 1H), 7.55 (d, *J* = 2.0 Hz, 1H), 6.90 (d, *J* = 8.4 Hz, 1H), 4.33 (s, 2H), 3.97 (s, 3H), 3.95 (s, 3H); IR (KBr): 2934, 2837, 1652, 1583, 1514, 1451, 1425, 1273, 1231, 1154, 1095, 1019, 877, 810, 768, 748, 617 cm^−1^; MS (ESI): 307.0 (M+H)^+^.

*2-Bromo-1-(3,4-dimethoxyphenyl)ethanone* (**12**). One mmol of substrate, 3 mmol IL-A, 1.1 mmol NBS, r.t. = 60 min, 55 °C, preparative chromatography SiO_2_, CH_2_Cl_2_, white solid (63%), mp: 80.5–81.6 °C (lit [63] 80–81 °C); ^1^H-NMR (CDCl_3_): δ 7.62 (dd, *J* = 8.4 Hz, *J* = 1.9 Hz, 1H), 7.55 (d, *J* = 1.9 Hz, 1H), 6.91 (d, *J* = 8.4 Hz, 1H), 4.41 (s, 2H), 3.97 (s, 3H), 3.95 (s, 3H); IR (KBr): 2966, 2940, 2843, 1682, 1586, 1515, 1466, 1420, 1283, 1244, 1155, 1020, 868, 798, 769, 683, 619 cm^−1^; MS (ESI): 259.0 (M+H)^+^, 261.0 (M+2+H)^+^.

*2,2-Dibromo-1-(3,4-dimethoxyphenyl)ethanone* (**13**). One mmol of substrate, 3 mmol IL-A, 2.2 mmol NBS, r.t. = 120 min, 70 °C; column chromatography SiO_2_, CH_2_Cl_2_; white solid (87%); mp: 79.0–81.7 °C; ^1^H-NMR (CDCl_3_): δ 7.74 (dd, *J* = 8.4 Hz, *J* = 2.0 Hz, 1H), 7.62 (d, *J* = 2.0 Hz, 1H), 6.92 (d, *J* = 8.4 Hz, 1H), 6.69 (s, 1H), 3.98 (s, 3H), 3.95 (s, 3H); ^13^C-NMR (CD_3_COCD_3_): 186.8, 156.9, 151.6, 126.2, 125.4, 113.7, 112.8, 57.4, 57.3, 43.3; IR (KBr): 3017, 2967, 2933, 2837, 1676, 1587, 1518, 1454, 1416, 1350, 1279, 1238, 1190, 1155, 1016, 881, 800, 756, 689, 621 cm^−1^; MS (ESI): 336.9 (M+H)^+^, 338.9 (M+2+H)^+^, 340.9 (M+4+H)^+^; HRMS: calcd for C_10_H_11_Br_2_O_3_: 336.9075; found: 336.9084.

*2-Iodo-1-(2,4-dimethoxyphenyl)ethanone* (**15**). One mmol of substrate, 3 mmol IL-A, 1.1 mmol NIS, r.t. = 10 min, 55 °C, preparative chromatography SiO_2_, CH_2_Cl_2_, brown solid (34%), mp: 64.5–67.0 °C (lit [48] 55–57.5 °C); ^1^H-NMR (CDCl_3_): δ 7.91 (d, *J* = 8.8 Hz, 1H), 6.56 (dd, *J* = 8.8 Hz, *J* = 2.3 Hz, 1H), 6.47 (d, *J* = 2.3 Hz, 1H), 4.46 (s, 2H), 3.94 (s, 3H), 3.87 (s, 3H); IR (KBr): 3009, 2940, 2838, 1657, 1595, 1499, 1454, 1414, 1333, 1267, 1211, 1161, 1109, 1023, 984, 826, 610 cm^−1^; MS *m/z* (CI): 307.0 (M+H)^+^.

*1-Acetyl-5-iodo-2,4-dimethoxybenzene* (**16**). One mmol of substrate, 3 mmol IL-A, 1.1 mmol NIS, r.t. = 10 min, 55 °C, preparative chromatography SiO_2_, CH_2_Cl_2_, grey solid (3%), mp: 130.3–134.0 °C (lit [48] 142–145 °C); ^1^H-NMR (CDCl_3_): δ 8.23 (s, 1H), 6.39 (s, 1H), 3.94 (s, 3H), 3.94 (s, 3H), 2.56 (s, 3H); IR (KBr): 1647, 1591, 1464, 1391, 1357, 1321, 1271, 1231, 1022, 830 cm^−1^; MS (ESI): 307.0 (M+H)^+^**,** HRMS: calcd for C_10_H_12_IO_3_: 306.9831; found: 306.9821.

*2-Iodo-1-(5-iodo-2,4-dimethoxyphenyl)ethanone* (**17**). One mmol of substrate, 3 mmol IL-A, 1.1 mmol NIS, r.t. = 10 min, 55 °C; preparative chromatography SiO_2_, CH_2_Cl_2_); yellow solid (8%); mp: 145.8–148.6 °C; ^1^H-NMR (CDCl_3_): δ 8.32 (s, 1H), 6.41 (s, 1H), 4.42 (s, 2H), 4.00 (s, 3H), 3.97 (s, 3H); ^13^C-NMR (CD_3_COCD_3_): δ 192.0, 165.2, 163.6, 143.4, 120.4, 98.1, 76.4, 58.4, 57.8, 10.7; IR (KBr): 1635, 1585, 1464, 1403, 1389, 1329, 1262, 1213, 1017, 919, 818 cm^−1^; MS (ESI): 432.9 (M + H)^+^; HRMS: calcd for C_10_H_11_I_2_O_3_: 432.8798; found: 432.8788.

*2-Iodo-1-(2,6-dimethoxyphenyl)ethanone* (**19**). One mmol of substrate, 3 mmol IL-A, 1.1 mmol NIS, r.t. = 10 min, 55 °C, preparative chromatography SiO_2_, CH_2_Cl_2_, yellow solid (47%), mp: 51.7–54.6 °C (lit [48] 58–60.5 °C); ^1^H-NMR (CDCl_3_): δ 7.31 (t, *J* = 8.4 Hz, 1H), 6.57 (d, *J* = 8.4 Hz, 2H), 4.30 (s, 2H), 3.83 (s, 6H); IR (KBr): 2937, 2837, 1713, 1593, 1474, 1429, 1378, 1307, 1284, 1250, 1161, 1121, 1091, 980, 773, 722 cm^−1^; MS (ESI): 307.0 (M+H)^+^.

*2-Iodo-1-(3-iodo-2,6-dimethoxyphenyl)ethanone* (**20**). One mmol of substrate, 3 mmol IL-A, 1.1 mmol NIS, r.t. = 10 min, 55 °C; preparative chromatography SiO_2_, CH_2_Cl_2_; yellow oil (10%); ^1^H-NMR (CDCl_3_): δ 7.75 (d, *J* = 8.8 Hz, 1H), 6.55 (d, *J* = 8.8 Hz, 1H), 4.31 (s, 2H), 3.84 (s, 3H), 3.82 (s, 3H); ^13^C-NMR (CD_3_COCD_3_): δ 195.4, 159.9, 159.0, 143.0, 125.0, 111.8, 82.0, 64.5, 57.9, 11.1; IR (NaCl): 2967, 2938, 2838, 1694, 1574, 1462, 1433, 1395, 1282, 1244, 1165, 1084, 1003, 913, 805 cm^−1^; MS (ESI): 432.9 (M + H)^+^: HRMS: calcd for C_10_H_11_I_2_O_3_: 432.8798; found: 432.8788.

*2,2-Dichloro-1-(3,5-dichloro-2,6-dimethoxyphenyl)ethanone* (**21**). One mmol of substrate, 3 mmol IL-A, 4.4 mmol NCS, r.t. = 1080 min, 85 °C; preparative chromatography SiO_2_, hexane/CH_2_Cl_2_ = 1/1; white solid (65%); mp: 53.4–54.4 °C; ^1^H-NMR (CD_3_COCD_3_): δ 7.77 (s, 1H), 6.92 (s, 1H), 3.92 (s, 6H); ^13^C-NMR (CD_3_COCD_3_): δ 189.0, 154.8, 135.5, 129.7, 125.4, 72.5, 64.3; IR (KBr): 3016, 2946, 1738, 1568, 1462, 1414, 1214, 1070, 984, 926, 872, 818, 773, 714, 641 cm^−1^; Anal. Calcd for C_10_H_8_Cl_4_O_3_: C, 37.77; H, 2.54. Found: C, 37.89; H, 2.36.

*1-Acetyl-3-bromo-2,6-dimethoxybenzene* (**22**). One mmol of substrate, 3 mmol IL-A, 1.1 mmol NBS, r.t. = 10 min, 55 °C, preparative chromatography SiO_2_, CH_2_Cl_2_, yellow oil [64] (63%); ^1^H-NMR (CDCl_3_): δ 7.48 (d, *J* = 8.9 Hz, 1H), 6.61 (d, *J* = 8.9 Hz, 1H), 3.83 (s, 3H), 3.81 (s, 3H), 2.49 (s, 3H); IR(NaCl): 3004, 2940, 2873, 2839, 1707, 1580, 1464, 1401, 1351, 1285, 1240, 1219, 1183, 1092, 1007, 971, 910, 801, 689, 652 cm^−1^; MS (ESI): 259.0 (M+H)^+^, 261.0 (M+2+H)^+^.

*2-Bromo-1-(3-bromo-2,6-dimethoxyphenyl)ethanone* (**23**). One mmol of substrate, 3 mmol IL-A, 1.1 mmol NBS, r.t. = 10 min, 55 °C; preparative chromatography SiO_2_, CH_2_Cl_2_; yellow oil (8%); ^1^H-NMR (CDCl_3_): δ 7.54 (d, *J* = 8.9 Hz, 1H), 6.64 (d, *J* = 8.9 Hz, 1H), 4.36 (s, 2H), 3.85 (s, 3H), 3.83 (s, 3H); ^13^C-NMR (CD_3_COCD_3_): δ 194.5, 158.9, 156.4, 137.1, 125.7, 111.1, 109.4, 64.1, 57.9, 38.6; IR (NaCl): 2941, 1707, 1580, 1464, 1403, 1285, 1227, 1180, 1132, 1088, 1005, 916, 803 cm^−1^; MS (ESI): 336.9 (M+H)^+^, 338.9 (M+2+H)^+^, 340.9 (M+4+H)^+^; HRMS: calcd for C_10_H_11_Br_2_O_3_: 336.9075; found: 336.9082.

*2,2-Dibromo-1-(3,5-dibromo-2,6-dimethoxyphenyl)ethanon*e (**24**). One mmol of substrate, 3 mmol IL-A, 4.4 mmol NBS, r.t. = 360 min, 85 °C; preparative chromatography SiO_2_, hexane/CH_2_Cl_2_ = 1/1); white solid (72%); mp: 54.9–57.6 °C; ^1^H-NMR (CD_3_COCD_3_): δ 8.05 (s, 1H), 6.88 (s, 1H), 3.90 (s, 6H); ^13^C-NMR (CD_3_COCD_3_): δ 188.5, 156.6, 140.9, 129.5, 114.4, 64.5, 45.2; IR (KBr): 3017, 2939, 1723, 1559, 1456, 1406, 1275, 1220, 1165, 1066, 982, 916, 799 615 cm^−1^; MS (ESI): 494.7 (M+2+H)^+^, 496.7 (M+4+H)^+^, 498.7 (M+6+H)^+^; HRMS: calcd for C_10_H_9_Br_4_O_3_: 492.7285; found: 492.7296; Anal. Calcd for C_10_H_8_Br_4_O_3_: C, 24.23; H, 1.63. Found: C, 24.37; H, 1.61.

*2-Iodo-1-(2,3,4-trimethoxyphenyl)ethanone* (**26**). One mmol of substrate, 3 mmol IL-A, 1.1 mmol NIS, r.t. = 60 min, 55 °C, preparative chromatography SiO_2_, CH_2_Cl_2_, yellow oil [63] (68%); ^1^H-NMR (CDCl_3_): δ 7.59 (d, *J* = 8.9 Hz, 1H), 6.73 (d, *J* = 8.9 Hz, 1H), 4.49 (s, 2H), 4.07 (s, 3H), 3.92 (s, 3H), 3.87 (s, 3H); IR (NaCl): 2941, 2840, 1661, 1587, 1493, 1464, 1410, 1287, 1212, 1096, 1005, 924, 881, 808, 697 cm^−1^; MS (ESI): 337.0 (M+H)^+^.

*2-Bromo-1-(2,3,4-trimethoxyphenyl)ethanone* (**27**). One mmol of substrate, 3 mmol IL-A, 1.1 mmol NBS, r.t. = 30 min, 55 °C, preparative chromatography SiO_2_, CH_2_Cl_2_, white solid [65] (44%), mp: 39.8–42.0 °C; ^1^H-NMR (CDCl_3_): δ 7.61 (d, *J* = 8.9 Hz, 1H), 6.74 (d, *J* = 8.9 Hz, 1H), 4.57 (s, 2H), 4.05 (s, 3H), 3.93 (s, 3H), 3.87 (s, 3H); IR (NaCl): 2941, 2843, 1680, 1589, 1493, 1464, 1410, 1294, 1213, 1099, 999, 809, 668 cm^−1^; MS (ESI): 289.0 (M+H)^+^, 291.0 (M+2+H)^+^.

*2,2-Dibromo-1-(2,3,4-trimethoxyphenyl)ethanone* (**28**). One mmol of substrate, 3 mmol IL-A, 1.1 mmol NBS, r.t. = 30 min, 55 °C; preparative chromatography SiO_2_, CH_2_Cl_2_; white solid (14%); mp: 42.9–44.6 °C; ^1^H-NMR (CDCl_3_): δ 7.64 (d, *J* = 9.0 Hz, 1H), 7.09 (s, 1H), 6.76 (d, *J* = 9.0 Hz, 1H), 4.08 (s, 3H), 3.94 (s, 3H), 3.87 (s, 3H); ^13^C-NMR (CD_3_COCD_3_): δ 188.2, 161.0, 155.5, 143.7, 128.8, 121.3, 109.9, 63.6, 62.0, 57.7, 46.5; IR (KBr): 3020, 2942, 1682, 1588, 1495, 1462, 1410, 1293, 1217, 1097, 989, 814, 687 cm^−1^; MS (ESI): 366.9 (M+H)^+^, 368.9 (M+2+H)^+^, 370.9 (M+4+H)^+^; HRMS: calcd for C_11_H_13_Br_2_O_4_: 366.9181; found: 366.9175; Anal. Calcd for C_11_H_12_Br_2_O_4_: C, 35.90; H, 3.29. Found: C, 36.29; H, 3.29.

*2-Bromo-1-(5-bromo-2,3,4-trimethoxyphenyl)ethanone* (**29**). One mmol of substrate, 3 mmol IL-A, 2.2 mmol NBS, r.t. = 120 min, 70 °C; preparative chromatography SiO_2_, CH_2_Cl_2_; colorless oil (37%); ^1^H-NMR (CDCl_3_): δ 7.75 (s, 1H), 4.52 (s, 2H), 4.05 (s, 3H), 3.99 (s, 3H), 3.90 (s, 3H); ^13^C-NMR (CD_3_COCD_3_): δ 191.6, 157.3, 155.8, 149.2, 129.8, 128.0, 113.0, 63.2, 62.5, 62.5, 38.6; IR(NaCl): 3073, 2942, 1692, 1574, 1462, 1403, 1304, 1260, 1223, 1092, 1051, 995, 932, 889, 872, 791, 674 cm^−1^; MS (ESI): 366.9 (M+H)^+^, 368.9 (M+2+H)^+^, 370.9 (M+4+H)^+^; HRMS: calcd for C_11_H_13_Br_2_O_4_: 366.9181; found: 366.9186; Anal. Calcd for C_11_H_12_Br_2_O_4_: C, 35.90; H, 3.29. Found: C, 35.91; H, 3.15.

*2,2-Dibromo-1-(5-bromo-2,3,4-trimethoxyphenyl)ethanone* (**30**). One mmol of substrate, 3 mmol IL-A, 3.3 mmol NBS, r.t. = 120 min, 70 °C; preparative chromatography SiO_2_, hexane/CH_2_Cl_2_ = 1/9); yellow oil (88%); ^1^H-NMR (CD_3_COCD_3_): δ 7.70 (s, 1H), 7.22 (s, 1H), 4.11 (s, 3H), 4.00 (s, 3H), 3.94 (s, 3H); ^13^C-NMR (CD_3_COCD_3_): δ 187.6, 157.8, 155.5, 149.0, 130.4, 125.3, 113.2, 63.7, 62.6, 62.6, 45.9; IR (NaCl): 2943, 1690, 1574, 1463, 1403, 1304, 1258, 1216, 1090, 1047, 993, 797, 669 cm^−1^; MS (ESI): 444.8 (M+H)^+^, 446.8 (M+2+H)^+^, 448.8 (M+4+H)^+^, 450.8 (M+6+H)^+^; HRMS: calcd for C_11_H_12_Br_3_O_4_: 444.8286; found: 444.8291; Anal. Calcd for C_11_H_11_Br_3_O_4_: C, 29.56; H, 2.48. Found: C, 29.44; H, 2.27.

*p-Iodoanisole* (**32a**). One mmol of substrate, 3 mmol IL-A, 1.1 mmol NIS, r.t. = 20 min, 55 °C, preparative chromatography SiO_2_, hexane/CH_2_Cl_2_ = 7.5/2.5, white solid (84%), mp: 42.1–44.4 °C (lit [48] 47–49.5 °C); ^1^H-NMR (CDCl_3_): δ 7.55 (d, *J* = 9.0 Hz, 2H), 6.68 (d, *J* = 9.0 Hz, 2H), 3.78 (s, 3H); IR (KBr): 2965, 2936, 2835, 1584, 1481, 1450, 1285, 1244, 1173, 1026, 993, 807 cm^−1^.

*p-Bromoanisole* (**32b**). One mmol of substrate, 3 mmol IL-A, 1.1 mmol NBS, r.t. = 20 min, 55 °C, CC (SiO_2_, hexane, colorless oil [9] (81%); ^1^H-NMR (CDCl_3_): δ 7.37 (d, *J* = 9.0 Hz, 2H), 6.78 (d, *J* = 9.0 Hz, 2H), 3.78 (s, 3H); IR (NaCl): 3004, 2957, 2937, 2903, 2835, 1579, 1487, 1460, 1290, 1249, 1171, 1072, 1032, 1003, 822 cm^−1^.

*p-Chloroanisole* (**32c**). One mmol of substrate, 3 mmol IL-A, 1.1 mmol NCS, r.t. = 30 min, 55 °C, preparative chromatography SiO_2_, hexane/CH_2_Cl_2_ = 7.5/2.5, colorless oil [66] (78%); ^1^H-NMR (CDCl_3_): δ 7.24 (d, *J* = 9.0 Hz, 2H), 6.83 (d, *J* = 9.0 Hz, 1H), 3.79 (s, 3H); IR (NaCl): 3006, 2959, 2940, 2906, 2837, 1593, 1492, 1460, 1290, 1245, 1181, 1169, 1092, 1063, 1032, 824, 750, 686, 639, 626 cm^−1^.

*2,4-Diiodoanisole* (**33a**). One mmol of substrate, 3 mmol IL-A, 2.2 mmol NIS, r.t. = 30 min, 70 °C, CC (SiO_2_, CH_2_Cl_2_, white solid (92%), mp: 65.8–67.0 °C (lit [67] 67.5–68.5 °C); ^1^H-NMR (CDCl_3_): δ 8.04 (d, *J* = 2.1 Hz, 1H), 7.58 (dd, *J* = 8.6 Hz, *J* = 2.1 Hz, 1H), 6.58 (d, *J* = 8.6 Hz, 1H), 3.86 (s, 3H); IR (KBr): 1582, 1469, 1429, 1275, 1246, 1182, 1142, 1034, 866, 797, 656 cm^−1^.

*2,4-Dibromoanisole* (**33b**). One mmol of substrate, 3 mmol IL-A, 2.2 mmol NBS, r.t. = 180 min, 85 °C, CC SiO_2_, hexane/CH_2_Cl_2_ = 7.5/2.5, white solid (90%), mp: 53.5–56.0 °C (lit [67] 61–62 °C); ^1^H-NMR (CDCl_3_): δ 7.67 (d, *J* = 2.3 Hz, 1H), 7.38 (dd, *J* = 8.8 Hz, *J* = 2.3 Hz, 1H), 6.77 (d, *J* = 8.8 Hz, 1H), 3.88 (s, 3H); IR (NaCl): 3080, 2973, 2936, 2835, 1574, 1476, 1456, 1379, 1289, 1250, 1052, 1020, 876, 804, 679, 619 cm^−1^.

*2,4-Dichloroanisole* (**33c**). One mmol of substrate, 3 mmol IL-A, 2.2 mmol NCS, r.t. = 30 min, 70 °C, CC SiO_2_, hexane/CH_2_Cl_2_ = 7.5/2.5, colorless oil [66] (89%); ^1^H-NMR (CDCl_3_): δ 7.37 (d, *J* = 2.5 Hz, 1H), 7.19 (dd, *J* = 8.8 Hz, *J* = 2.5 Hz, 1H), 6.85 (d, *J* = 8.8 Hz, 1H), 3.89 (s, 3H); IR (NaCl): 3075, 3011, 2965, 2940, 2901, 2841, 1580, 1484, 1439, 1389, 1292, 1263, 1184, 1106, 1065, 1025, 870, 835, 805, 716, 642 cm^−1^.

*1,5-Diiodo-2,4-dimethoxybenzene* (**35a**). One mmol of substrate, 3 mmol IL-A, 2.2 mmol NIS, r.t. = 20 min, 70 °C, column chromatography (SiO_2_, CH_2_Cl_2_, white solid (89%), mp: 193.6–196.5 °C (lit [68] 201–202 °C); ^1^H-NMR (CDCl_3_): δ 8.04 (s, 1H), 6.37 (s, 1H), 3.89 (s, 6H); IR (neat): 1568, 1556, 1458, 1452, 1428, 1357, 1275, 1207, 1177, 1034, 1014, 886, 813, 652 cm^−1^; MS (ESI): 389.9 (M)^+^.

*1,5-Dibromo-2,4-dimethoxybenzene* (**35b**). One mmol of substrate, 3 mmol IL-A, 2.2 mmol NBS, r.t. = 30 min, 70 °C, preparative chromatography SiO_2_, hexane/CH_2_Cl_2_ = 2.5/7.5, white solid (70%), mp: 135.5–138.4 °C (lit [69] 138–139 °C); ^1^H-NMR (CDCl_3_): δ 7.66 (s, 1H), 6.50 (s, 1H), 3.91 (s, 6H); IR (neat): 1579, 1570, 1487, 1463, 1432, 1367, 1287, 1208, 1175, 1061, 1052, 1019, 877, 864, 813, 805, 687, 680 cm^−1^.

*1,5-Dichloro-2,4-dimethoxybenzene* (**35c**). One mmol of substrate, 3 mmol IL-A, 2.2 mmol NCS, r.t. = 120 min, 70 °C, preparative chromatography SiO_2_, hexane/CH_2_Cl_2_ = 2.5/7.5, white solid (82%), mp: 111.6–114.2 °C (lit [70] 118–119 °C); ^1^H-NMR (CDCl_3_): δ 7.36 (s, 1H), 6.54 (s, 1H), 3.91 (s, 6H); IR (neat): 1738, 1578, 1492, 1466, 1454, 1428, 1374, 1295, 1229, 1206, 1171, 1085, 1021, 861, 804, 740 cm^−1^.

*2,4,4-Trichloro-5-methoxycyclohexa-2,5-dienone* (**36**). One mmol of substrate, 3 mmol IL-A, 3.3 mmol NCS, r.t. = 45 min, 70 °C; preparative chromatography SiO_2_, hexane/CH_2_Cl_2_ = 2.5/7.5; yellow solid (84%); mp: 114.6–116.2 °C; ^1^H-NMR (CDCl_3_): δ 7.13 (s, 1H), 5.65 (s, 1H), 3.94 (s, 3H); ^13^C-NMR (CD_3_COCD_3_): δ 178.3, 169.5, 139.7, 132.4, 101.2, 76.3, 59.3; IR (neat): 1738, 1657, 1595, 1459, 1347, 1269, 1217, 1156, 1034, 974, 934, 898, 838, 783, 698, 686, 608 cm^−1^; MS (ESI): 226.9 (M+H)^+^, 228.9 (M+2+H)^+^, 230.9 (M+4+H)^+^; HRMS: calcd for C_7_H_6_Cl_3_O_2_: 226.9433; found: 226.9431; Anal. Calcd for C_7_H_5_Cl_3_O_2_: C, 36.96; H, 2.22. Found: C, 37.13; H, 2.23.

*p-Iodotoluene+o-Iodotoluene* (**38a**, **39a**). One mmol of substrate, 3 mmol IL-A, 1.1 mmol NIS, r.t. = 20 min, 55 °C, preparative chromatography SiO_2_, hexane/CH_2_Cl_2_ = 99.7/0.3, yellow oil [71] (49%); ^1^H-NMR (CDCl_3_): δ 7.80 (d, *J* = 7.8 Hz, 1H), 7.56 (d, *J* = 8.1 Hz, 2H), 7.23 (d, *J* = 4.1 Hz, 2H), 6.92 (d, *J* = 8.1 Hz, 2H), 6.90-6.82 (m, 1H), 2.43 (s, 3H), 2.29 (s, 3H).

*p-Bromotoluene+o-Bromotoluene* (**38b**, **39b**). One mmol of substrate, 3 mmol IL-A, 1.1 mmol NBS, r.t. = 20 min, 55 °C; ^1^H-NMR (CDCl_3_): δ 7.55–7.49 (m, 1H), 7.36 (d, *J* = 8.3 Hz, 2H), 7.29–7.14 (m, 2H), 7.08–6.99 (m, 3H), 2.40 (s, 3H), 2.30 (s, 3H) [72].

*p-Chlorotoluene+o-Chlorotoluene* (**38c**, **39c**). One mmol of substrate, 3 mmol IL-A, 1.1 mmol NCS, r.t. = 180 min, 70 °C; ^1^H-NMR (CDCl_3_): δ 7.37–7.31 (m, 1H), 7.25–7.07 (m, 7H), 2.39 (s, 3H), 2.32 (s, 3H) [73].

*5-Iodo-2-methylthiophene* (**41**). One mmol of substrate, 3 mmol IL-A, 1.1 mmol NIS, r.t. = 10 min, 55 °C, CC SiO_2_, hexane/CH_2_Cl_2_ = 7.5/2.5), yellow oil [74] (81%); ^1^H-NMR (CDCl_3_): δ 7.00 (d, *J* = 3.5 Hz, 1H), 6.44 (dq, *J* = 3.5 Hz, *J* = 0.9 Hz, 1H), 2.49 (d, *J* = 0.9 Hz, 3H).

### 3.3. Scale up Experiment

To a stirred mixture of acetophenone (**1**, 9 mmol, 1.08 g) in IL-A (27 mmol, 9.95 g), *N*-bromo succinimide (19.8 mmol, 3.52 g) was added. The reaction mixture was stirred at 70 °C for 40 min. Then the mixture was cooled to room temperature and washed three times with dichloromethane. The combined organic fractions were washed with aqueous Na_2_S_2_O_3_ and NaHCO_3_, dried over anhydrous Na_2_SO_4_ and filtered. Solvent was removed under reduced pressure and the crude reaction mixture was analyzed with ^1^H-NMR. Purification of crude reaction mixture by chromatography yielded 2.25 g (90%) of **3b**.

### 3.4. Recycling/Reuse of IL-A in Iodination of Acetophenone *(**1**)* with NIS

To a stirred mixture of acetophenone (**1**, 1 mmol, 120 mg) in IL-A (3 mmol, 368 mg), *N*-iodo succinimide (1.1 mmol, 225 mg) was added. The reaction mixture was stirred at 55 °C for 10 minutes. Then the mixture was cooled to room temperature and washed three times with dichloromethane. The combined organic fractions were washed with aqueous Na_2_S_2_O_3_ and NaHCO_3_, dried over anhydrous Na_2_SO_4_ and filtered. Solvent was removed under reduced pressure and the crude reaction mixture was analyzed with ^1^H-NMR. Purification of crude reaction mixture with chromatography yielded 212 mg (86%) of **2a**. The IL-A from reaction was dried under reduced pressure and reused.

## 4. Conclusions 

In summary, the Brønsted acidic ionic liquid 1-methyl-3-(4-sulfobutyl)imidazolium triflate ([BMIM(SO_3_H)][OTf]; IL-A) exhibited a notable catalytic effect in the halofunctionalization of aromatics with NXS and transformations were significantly faster as compared to IL-B. Aryl methyl ketones were α- and α,α-dihalogenated with high selectivity and in respectable yields. In the case of methoxy-substituted acetophenones, competing ring halogenation occurred. Activated arenes were selectively mono- and dihalogenated. A noteworthy feature is exclusive *para*-halogenation of anisole with NXS/IL-A. Recycling and reuse of [BMIM(SO_3_H)][OTf] was demonstrated in a prototype iodination reaction in eight cycles with no decrease in the conversion. The catalytic role of IL-A in dibromination with NBS is manifested in higher mono- to dihalogenation ratios when the reaction is repeated in the used IL. The feasibility for scale-up was also demonstrated in a representative case. 

## Figures and Tables

**Table 1:**
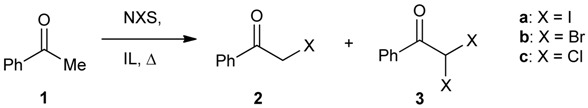
Halogenation of acetophenone **1** with *N*-halosuccinimides (NXS) in 1-butyl-3-methylimidazolium based ionic liquids ^a^.

Entry	X	NXS (equiv.)	IL	T (°C)	Reaction time (min)	Conversion of 1 ^b^ (%)	2 / 3
1	I	1.1	IL-B	55	10	13	100 / 0
2	I	1.1	IL-A	55	10	98	93 / 7
3	Br	1.1	IL-B	55	20	22	100 / 0
4	Br	1.1	IL-A	55	20	95	86 / 14
5	Br	2.2	IL-B	70	30	60	100 / 0
6	Br	2.2	IL-A	70	30	100	0 / 100
7	Cl	1.1	IL-B	70	30	97	85 / 15
8	Cl	1.1	IL-A	70	30	47	90 / 10
9	Cl	1.1	IL-A	70	90	63	15 / 85
10	Cl	2.2	IL-B	70	30	92	91 / 9
11	Cl	2.2	IL-A	70	30	100	0 / 100

^a^ *Reaction conditions*: 1 mmol of **1**, 1.1 or 2.2 mmol of NXS stirred in 3 mmol of IL; ^b^ Conversion determined by ^1^H-NMR.

**Table 2:**
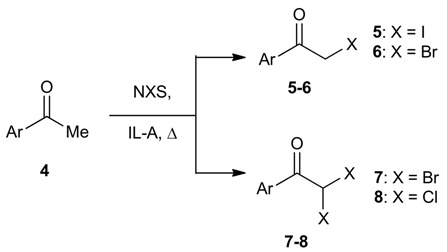
The effect of aryl methyl ketone **4** structure on halofunctionalization with *N*-halosuccinimides (NXS) in [BMIM(SO_3_H)][OTf] (IL-A) ^a^.

Entry	Ar	Reaction conditions^a^ NXS (mmol) / T (°C) / t (min)	Products	Yield (%) ^b^	
				5 or 6	7 or 8
1	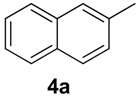	NIS (1.1) / 55 / 10	**5a**	94 (84)	
2		NBS (1.1) / 55 / 20	**6a / 7a**	72 (66)	19 (18)
3		NBS (2.2) / 70 / 60	**7a**		96 (94)
4		NCS (2.2) / 70 / 150	**8a**		97 (86)
5	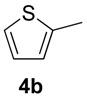	NIS (1.1) / 55 / 10	**5b**	91 (80)	
6		NBS (1.1) / 55 / 10	**6b / 7b**	75 (72)	16 (15)
7		NBS (2.2) / 70 / 30	**7b**		95 (85)
8		NCS (2.2) / 70 / 60	**8b**		96 (67)
9	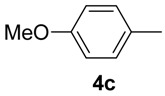	NIS (1.1) / 55 / 10	**5c**	92 (80)	
10		NBS (1.1) / 55 / 20	**6c / 7c**	71 (62)	22 (20)
11		NBS (2.2) / 70 / 30	**7c**		100 (91)

^a^ *Reaction conditions*: 1 mmol of **4**, 1.1 or 2.2 mmol of NXS stirred in 3 mmol of IL-A at 55–70 °C for 10 to 150 min; ^b^ Relative yields determined by ^1^H-NMR, values in the brackets refer to the yields of isolated pure products.

**Table:**
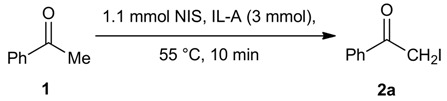


Repetition number	Yield (%)
1	86
2	90
3	90
4	88
5	90
6	89
7	87
8	92

**Table:**
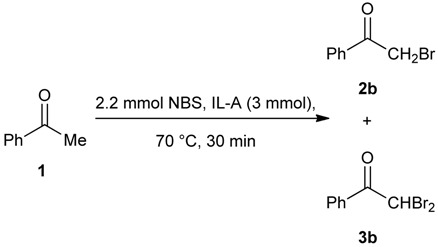


Repetition number	Product distribution (2b:3b)
1	0:100
2	4:96
3	12:88
4	25:75
5	33:67

## Figures, Schemes and Tables

Table 3 The effect of [BMIM(SO_3_H)][OTf] (IL-A) recycling on transformation of acetophenone **1** with NXS reagents.
